# The Whole of a Scientific Career: An Interview with Oliver Smithies

**DOI:** 10.1371/journal.pgen.1005224

**Published:** 2015-05-28

**Authors:** Jane Gitschier

**Affiliations:** Departments of Medicine and Pediatrics and Institute for Human Genetics, University of California San Francisco, San Francisco, California, United States of America

They say that the third time’s the charm, and in the case of my interviewing Oliver Smithies (Fig 1), this old saw held true. Twice before over the past five years I had scheduled an interview with Smithies, only to find myself canceling for one reason or another. Fortunately, Smithies didn’t hold my fickleness against me and agreed to an interview in mid-February. I plotted my travel to Chapel Hill (where Smithies is on the faculty of the University of North Carolina) along a southern route to avoid the blizzards that paralyze the Chicago, Denver, and New York airports every winter, only to encounter an ice-storm that sent the Research Triangle into its own deep freeze. Fortunately, my plane and I made it safely, and I had no trouble meeting Smithies at the appointed time.

**Fig 1:**
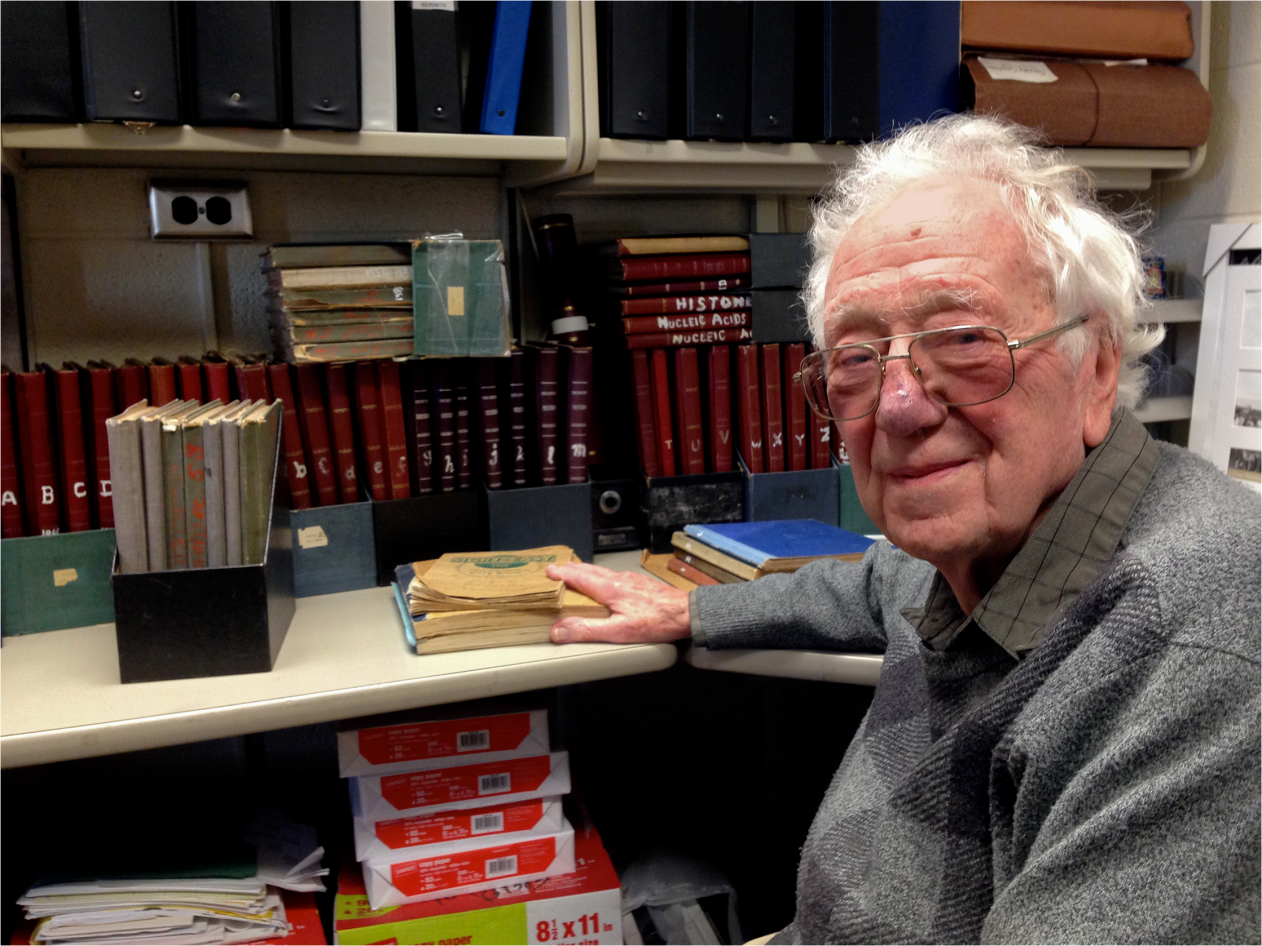
Oliver Smithies with a lifetime of notebooks.

Smithies, of course, is well worth any pilgrimage. Nearing 90 years of age, he still works at the bench, seven days a week. He is enthusiastic, curious, gentle, and fearless in attacking new problems, to which he applies his gifts both as a tinkerer and a thinker. He is generous with his ideas and advice and beloved by his colleagues, students, and postdocs, now numbering so many that he has lost count. His scientific journey began in the mid-late 1940s as an undergraduate at Balliol College, Oxford, where his tutor introduced him to a new field, now called “molecular biology.” Smithies embraced the young field, and after a brief postdoctoral stint at University of Wisconsin, took his first job in Toronto. There, in the early 1950s, he invented starch gel electrophoresis, which had the property of fractionating proteins on the basis of size and led him to discover inherited differences in haptoglobin, a serum protein that binds hemoglobin. One variant, the product of an abnormal genetic exchange, piqued his life-long interest in homologous recombination. Three decades later, after an arduous, three-year experiment, he was able to demonstrate homologous recombination between a plasmid and the human genome in the pursuit of correcting genetic defects, a discovery for which he, much later, won the Nobel Prize.

I got off the elevator to the seventh floor of the Brinkhous-Bullitt Building to find the hallway walls plastered with photos from the Smithies lab, as well as that of his wife, Nobuyo Maeda, with whom he often collaborates. Smithies led me through a tiny and fabulously cluttered office, which proved to be Maeda’s, and we then settled into *his* office, the lab itself. Smithies began the interview first with a few insights about my work and a compliment to our journals.

**Smithies:** Well, away we go then! Incidentally, I must say that I admire PLOS.

**Gitschier:** Oh, because of the open access?

**Smithies:** I think that is grand! And it is a good idea to have a place where the work is looked at by reviewers who do not have a stereotyped view that the paper should fit this or that sort of pattern. Where it can be small or it can be big and it won’t matter, because in the end, what matters is that it be available for others to read and that it should get into PubMed.

**Gitschier:** Well, thank you. I’ve enjoyed working with them.

You’ve had the longest research career of any person I have interviewed, and I get the sense, from reading your Nobel Prize speech and other articles, that you have kept all of your notebooks. I was curious about them; I noticed some are labeled with Greek letters, while others with Roman numerals.

**Smithies:** Well, the labeling is sort of random. I started with Roman numerals, and I got up to about 30. I even have my lab notebooks from when I was a graduate student.

**Gitschier:** I think you also have a paper from when you were an undergraduate, don’t you? That was published in ‘48.

**Smithies:** That was a paper with Sandy Ogston, and I was sort of an honorary author. At Oxford we had weekly tutorials, and you read to your professor an essay that you had written on whatever topic had been assigned to you the week before. Sandy said, “Write me an essay, Oliver, on energy metabolism.” Now this was before Krebs cycle was worked out, although [Severo] Ochoa had done some things that looked a bit cyclic.

I worked out a cycle that would take inorganic phosphate and make an ester out of it, which does not take much energy, and then by removing some hydrogen, I would end up with ATP. Then I could go around by giving the hydrogen back. So I developed a cycle that would convert inorganic phosphate to ATP indefinitely! It was obvious that it was wrong, you can’t get energy for nothing! But it wasn’t obvious *why* it was wrong.

It actually took quite a long time to figure out why it was wrong and what it meant. In the end, it turned out that it was a matter of concentrations, because at that time all energy calculations assumed that the concentrations of reactants and products were 1 M. It didn’t take into account what the real concentrations were. Sandy also worked out that oxidative metabolism couldn’t possibly function properly if the substrates dissociated from the enzymes, because their concentrations would be so low that the reactions wouldn’t take place fast enough. So he postulated that there had to be some sort of complex in which the substrates were kept bound to the enzymes, and of course that turns out to be true!

**Gitschier:** Now, you didn’t really have a lab notebook then, when you were an undergraduate, because you didn’t do any experimental work?

**Smithies:** No, but I still have some of those essays! A few of them that I enjoyed.

**Gitschier:** Do you have plans for a place where all of your work will be archived?

**Smithies:** Well, I’m working on it. My old college, Balliol, got me started with a small gift of money to have them electronically scanned commercially, but that didn’t work out, and I was going to have them electronically archived through the University here in North Carolina. But they came across the name of a person, and they said, “We can’t do it!”

**Gitschier:** What do you mean?

**Smithies:** Well, 50 years ago [when Smithies worked in Toronto] I tested blood samples from a family with a diabetic child, and so their name is written in my notebook, and we don’t have permission to use this family’s medical history! So they stopped the scanning!

**Gitschier:** Oh, that is ridiculous!

**Smithies:** Absolutely ridiculous! They stopped like that. So we’re doing it ourselves, by copying the notebooks, page by page. Some are very fragile. They are old and on poor paper. We’ve got probably fifty of them done. And I’m just arranging with my long-time North Carolina secretary [Jenny Holt], who worked with us for 25 years, to come back and we’re going to finish it. I’m intending to spend time with her voicing over the notebooks, describing what was going on at the time each page was written.

**Gitschier:** What are you planning to do with the physical notebooks?

**Smithies:** Well, I haven’t decided. They might go to my old college in Oxford, because they are interested in archiving things, and they started the thought. I’m not being vain when I say that it’s a rather unusual archive, because it has the whole of a scientific career.

**Gitschier:** Absolutely. And most peoples’ careers aren’t this long!

**Smithies:** That’s right! I’ll give you an idea about what I’m doing today, although I haven’t done experiments for maybe a month because I’ve been writing some papers. Here we are in February. Here’s what I’m writing today: it’s about the work that Jen Wilder, my technician, has been doing with her TEM [transmission electron microscope]. I’m also writing down what I’m thinking about because, obviously, when you get old your short-term memory gets much poorer. My way around that problem is to write down what I’m thinking about, so it won’t matter that tomorrow I will have forgotten what I was thinking about today.

**Gitschier:** Do you know of other scientists who have worked at the bench this late in their lives?

**Smithies:** It’s sort of unusual. I was at the beginning of some exciting molecular things. For example, I was a student at the time when scientists realized that proteins are giant molecules, not large aggregates of small clusters of amino acids.

**Gitschier:** Your first notebook, you think, was as a graduate student in Oxford?

**Smithies:** Yes, I have my graduate student notebooks. Let’s just see.

**Gitschier:** I’m just going to walk in here with you.

[We move back through the cluttered office and into an anteroom with bookshelves stacked with Smithies’ old notebooks.]

**Smithies:** I never thought of them as being important except for me to use in my everyday life, to go back and refer to them. They weren’t kept with any view of anybody ever wanting to see them except me!

**Gitschier:** Those look really old. [Pointing at one set.]

**Smithies:** Well, they *are* old! [Laughter.] These are from my first job in Toronto; they use Roman numbers. And these are my planning notes from 1953 [a small paperback].

**Gitschier:** Oh, this is amazing.

**Smithies:** You can see that it is the equivalent of a Google search. I went to the collection of chemical abstracts and looked up all the references that included the word “insulin,” and here are all the references that had to be checked.

**Gitschier:** Got it.

**Smithies:** So here we are; I knew I had my graduate student stuff somewhere! Here’s my thesis, and here are the four books from my graduate work [numbered 1, 2, 3, 4].

So this is “1948, O. Smithies, Balliol College.” [He leafs through it.] There is the first page, the circuit for a thermostat. That was the first thing that I had to make. Developing the water bath and doing some measurements and this, that, and the other. There is my home address [in Halifax, England].

Somewhere I have the notebooks from my postdoc years [probably in a box under the bookshelves].

**Gitschier:** What happened after the Roman numerals?

**Smithies:** Then I began with letters of the alphabet, A, B, C, D, etc. And then I went from capital letters to small letters. And then I went to Greek letters, I think, and then I went to A′, B′, C′, then a′, b′, c′, etc. [Laughter.] The labeling is not *very* systematic.

**Gitschier:** How many total do you think you have at this point?

**Smithies:** Oh, about 150-something, I think.

[As we re-enter the lab, I spy an old photo of two boys, dressed alike.]

**Gitschier:** Is this you and your brother [a fraternal twin]?

**Smithies:** Yeah. Which is which?

**Gitschier:** This is you [the one on the right, with the smiling face]!

**Smithies:** Yes, that’s right. When we came back from having that school picture taken, we were about seven years old, and my brother, Roger, said, “You know that Oliver didn’t have his coat buttoned up and his cap was on crooked.” [Laughter.] I had the happy genes, I always said.

In Toronto, that’s when I became a geneticist. I invented starch gel electrophoresis and with it found variants [in haptoglobin]. I also found a good geneticist, Dr. Norma Ford Walker, a marvelous person. And she taught me genetics. I really became a molecular geneticist at that point, and I moved back to Wisconsin.

**Gitschier:** The haptoglobin variants really initiated your interest in homologous recombination. Let’s talk more about that.

**Smithies:** Haptoglobin proved to have two polypeptide chains: alpha and beta [produced as a single polypeptide, and then clipped]. Alpha was small and beta was big. All the variations we saw were in the alpha chain.

What we found out was—and it took us a long time to find this out—that there are two primary forms of the alpha chain. We called them “Hp1” and “Hp2.” Hp2 form was bigger than Hp1; in fact, we later found out it was in fact a partial duplication of Hp1.

**Gitschier:** And there are also two versions of Hp1—called fast and slow—because they have some amino acid differences.

**Smithies:** Yeah, but we didn’t know that at first; we just knew 1 and 2, and then later we subdivided 1 into 1F and 1S for fast and slow.

**Gitschier:** And then you hypothesized at some point that this Hp2 was actually produced by a rare, non-homologous recombination.

**Smithies:** I remember exactly what happened.

By that time, I was back in Wisconsin, and my two collaborators, George Connell and Gordon Dixon, were still in Toronto. I remember very distinctly going back to visit them in Toronto, and we were trying to understand our results. Nobody had yet learned to do amino acid sequencing, but it was possible to look at peptides. There was a peptide characteristic of 1F and another characteristic of 1S, and for some crazy reason, the gene 2 chain had both 1F and 1S peptides, and it had another peptide, too, which eventually was known as the junction peptide, but we didn’t call it that at first. We called it “the 2 peptide in the 1 position,” or crazy names like this. We just didn’t know what it was.

I was getting ready for a talk, in Japan I think, and looking at the peptide data we had, and I said, it’s strange that this single, extra peptide has the same amino acids as the sum of these two. We couldn’t believe our results! We kept thinking that Hp1F and Hp1S were derived from Hp2 by proteolysis or something like that.

And I remember saying when I was there in Toronto, “Let’s see what happens if we *believe* our results!” And I suddenly realized that Hp2 was like a junction of Hp1F and Hp1S because I could see that two peptides, which we eventually knew to be the N- and C-terminal peptides, were present in the gene 2 product at half the concentration per mg of Hp2 protein as they were in the Hp1 product. So Hp2 is twice as long as Hp1! And all of our results were understandable!

So I came back from Toronto, and I went to Jim Crow [who was head of Medical Genetics and Genetics in Wisconsin]. I said to Jim, “How can I take two genes that are separate and join them together?” And he said, “Oh, that is well understood!”

He told me what was known about the *Drosophila* Bar locus. The Bar mutant [which reduces the size of the fly’s eyes] is a duplication. It has two big chunks of DNA, not just a single gene, and the duplication is big enough to see as bands in a *Drosophila* polytene chromosome. The original Bar locus mutation occurred only *once*: it was a non-homologous crossing-over event that led to a duplication. Now once you’ve got a duplication, you can easily go from two to three, and you can also go back to one by homologous recombination, and these events are *predictable*.

So I went back to the literature and found the history of the Bar locus, and understood that the Bar mutation and the formation of the Hp2 gene were both due to non-homologous recombination—and only arose once.

**Gitschier:** And of course, we now know that homologous recombination of the haptoglobin 2 can expand to a triplication, just as with the Bar locus. In fact, non-homologous and homologous recombination events are the basis for so much of human inherited disease.

I want to touch a little bit on immunoglobulins because you spent a lot of time thinking about how they might arise via homologous recombination, too, in the 1960s and 1970s.

**Smithies:** People were beginning to understand what the structure of an immunoglobulin was. They knew that there was a constant part and a variable part; Rodney Porter was the most important person in getting to that. And if you cut with a certain enzyme, papain I think it was, you could separate the variable and constant parts, and the constant part was so constant that it would crystallize.

It’s funny that when Rodney Porter got the Nobel Prize for that work, I happened to be purifying immunoglobulins, and following his work I had cut my protein with papain, and when I came back the next morning there were crystals in my test tube! So I knew that he had probably crystallized the constant part quite accidentally! I sent him a letter, but he never did reply.

Not long after this work, people were starting to do amino acid sequencing of the variable part, and Leroy Hood, who was a graduate student in William Dreyer’s lab, had sequenced the variable part on a number of myeloma proteins: the Bence Jones proteins, the light chains of the immunoglobulin.

**Gitschier:** So different patients had different Bence Jones proteins, so you’d collect urine, and sequence.

**Smithies:** Yes, that’s right. It was still difficult to do sequencing, but it was possible. I went to a meeting, probably at Cold Spring Harbor, and Leroy Hood talked about his results, and I asked if I could use them. His response was very kind: “You can use my data; you can think about it and do what you want with it.” So I took his sequences, and I tried to work out what was changing and came up with the idea that maybe it was due to crossing over. If you had a master gene, as it were, and another gene like it but not identical, and then you crossed over between the two, you could scramble the two sequences and get all sorts of different combinations of them.

I published a hypothesis, in *Nature* I think, on trying to understand antibody variation in terms of recombination between two genes.

It turns out that that is not how it works in humans, although I have a vague memory that it works that way in some other species—birds I think.

**Gitschier:** And then you had another model. I think we called it the train-track model [published in *Science*, 1970].

**Smithies:** Yes, that’s right. Well, I thought there would be multiple genes in parallel and then you could just transcribe one of the many possible tracks.

**Gitschier:** Now, did you actually do much experimental work on immunoglobulins?

**Smithies:** Oh yes, we worked out how to sequence them automatically. In those days, the way people were doing amino acid sequencing—the way Nobuyo was doing it—was very laborious. It was the Edman degradation method [invented in 1950] carried out manually.

Then Edman and Begg [1967] came up with an automatic machine for doing protein sequence that would carry out the Edman degradation in one step and then wash things out with a spinning cup and then do the next step. And you would go step by step by step, and collect what you got each time. And say the amino acids were ABCD. You’d get A the first time, B the second, but it wasn’t perfect. So you’d get 90% of A [in the first step] and then next you’d get 90% of B plus 5% of A, and then C you’d get 90% of C plus a little bit of B and less of A, and so it was complicated. We had to work out a computer program that would correct these things, by adding back what was carry-over. We actually got the machine to work very well and to do many amino acids. I think I got 110 amino acids in one run. It was very powerful, and the paper got quoted a lot, probably because of the computer program. Anyway, that was the sequenator.

**Gitschier:** So this would have been probably in the ‘70s.

**Smithies:** Don’t ask me when it was!

Let me return to the branched/train-track model. It predicted a specific DNA structure. So I started to learn how to handle DNA to see if I could see things like that in the electron microscope!

**Gitschier:** Oh my, from human cells?

**Smithies:** Well, actually, I wanted to see if I could *make* branched DNA! I never published the experiments, but I did publish the hypothesis. “Pathways Through Networks of Branched DNA.” That’s what it was.

I went to Bill Dove’s lab [also at Wisconsin] and learned how to make bacteriophage lambda. And lambda dv was a very small plasmid that came from lambda and that could replicate. People weren’t yet doing much work with plasmids, and lambda dv was one of the few pieces of DNA available at that time which was circular and could be purified. So I thought I would try to make that structure out of the DNA.

**Gitschier:** Wow.

**Smithies:** Well, I never did succeed.

After [Susumu] Tonegawa’s discovery, I decided that the guts of understanding immunoglobulin variability had been already captured. I wasn’t really interested in going on with the immunoglobulin work, and I decided to work with hemoglobin genes, which was an obvious choice. I didn’t have any particular goal, except that these genes were obviously interesting and tackle-able, and I wanted to do DNA work.

**Gitschier:** Of course you went on to work on Hemoglobin Lepore [another case of recurrent homologous recombination in humans].

When did you start thinking about repairing a defective sickle cell? I’m interested in the genesis of that idea.

**Smithies:** Well, I was teaching a molecular genetics course, so I knew about Terry Orr-Weaver’s work in yeast, where you could get homologous recombination [1981]. She could get a plasmid to integrate into a chosen yeast gene. So we knew that homologous recombination between a plasmid and a gene was possible in yeast and at very high frequency.

**Gitschier:** And had you ever thought before her work that you might imagine doing this in a human cell?

**Smithies:** No, I probably didn’t. Almost certainly the idea came from knowing Terry’s work. I knew about homologous recombination, but the yeast genome is so tiny compared with the human genome that I think a lot of people thought it wouldn’t be possible in mammals.

I don’t know when I got to thinking about correcting the sickle cell mutation—it was obviously something people were thinking about. You have the DNA for the gene, and you know what is wrong—so it was obvious that, if you could get it to work, it would be useful. But Terry Orr-Weaver’s work was inspirational. If you look at my paper, I’m sure I say that at the very beginning.

**Gitschier:** You do. One of your collaborators at the time was Raju Kucherlapati. How did you get to know him?

**Smithies:** Well, he and I got to know each other a long time earlier. He was in the lab of the person who was mapping genes on chromosomes.

**Gitschier:** Frank Ruddle?

**Smithies:** Yes. Ruddle. Raju was mapping the beta-2 microglobulin gene, and I had been the first person to publish any sequence from beta-2 microglobulin. And somehow we got in touch with each other as a result of that. Raju and I had also done work together with Art Skoultchi [at Albert Einstein] on things related to hemoglobin.

When I began to think about this [homologous recombination experiment], I needed someone to do the culture work, and Raju was doing cultures with human cells. He was then in Chicago. We decided to collaborate again; I would make the targeting DNA, and he would test it in cell cultures. Making the targeting vector in those days was quite difficult because we didn’t have things like linkers, you couldn’t buy stuff, you couldn’t synthesize DNA. But I started to make the vector, and eventually I got it.

Meanwhile, Raju got the same cells that had been used in a 1982 paper from Michael Wigler’s lab that gave me the idea of how to do the experiment. My strategy involved the same strategy that they used; it involved getting a small piece of DNA, a suppressor of amber mutations, supF. If you had an amber mutant in the bacteriophage, unless it picked up the supF, it wouldn’t grow. Therefore, I had a tool that would capture any DNA that included supF [in the library that would be made from the human cell line DNA].

Then I had to have something that would recognize my target—the beta-globin gene. Because of work Fred Blattner and I had done together in the late 1970s, I knew that you can find a bacteriophage which has the hemoglobin gene in it by hybridizing with a radioactive probe. I still have the films from those days! They were big—huge—like this. [Gesturing.] We would plate bacteria phages in cafeteria trays, not in little Petri dishes.

So, when the bacteriophages are growing, you know they must have got supF. Now the question is have they got the beta globin gene as well? Well you can answer this question by hybridizing with a radioactive probe, looking for the two [supF and the beta globin gene] coming together in what we called the recombinant fragment.

**Gitschier:** Right, so that is the genesis of this 1985 paper. This was very cumbersome, this technique.

**Smithies:** Oh, extremely!

**Gitschier:** And then PCR was developed.

**Smithies:** PCR got rid of all the bacteriophage work, because now you could find the recombinant fragment with PCR. And over there is my homemade PCR machine that was used for the experiment. Do you want to see it?

**Gitschier:** You still have it? The “hexapus,” right?

**Smithies:** It was working up until recently, when it developed an air leak. [We move across the hall.] This is it. We called it hexapus because it has six tentacles.

**Gitschier:** I had no idea it was so big!

**Smithies:** Well, yes. I made it from old stuff. These are all old discarded things. You couldn’t *buy* a PCR machine until one or two years later.

I’m fortunate in never being afraid of trying something new. So, for example, in the work I’m doing with the kidney, I needed electron microscopy. I found it took three months to get an electron micrograph done through the department. I got so fed up with that that we learned to do our own electron microscopy, and now we do all our own work. And what I’ve been doing today is getting together all the images we have to analyze with respect to the kidney. These are *our* electron micrographs, you see, taken by my technician, Jen Wilder, starting with freshly excised kidneys.

**Gitschier:** That is amazing, honestly. When you look back on this obviously very remarkable career, what were your favorite experiments, or discoveries, or favorite times?

**Smithies:** Well, I always show that page in my notebook where I wrote down the scheme to find out if homologous recombination could work.

**Gitschier:** I know that page, yes. I saw it in your Nobel Prize speech.

**Smithies:** That is the most important page in all my notebooks, I think.

What was the most enjoyable experiment? I suppose seeing that it worked, which was three years later! Developing the film and seeing the right spot, that we really had targeted the globin gene.

**Gitschier:** Well, nowadays, with people using things like CRISPR, things are so quick.

**Smithies:** Oh, I know, it’s marvelous. I’m delighted to see it, because the old method was very laborious. I think CRISPR opens the possibility that people will be able to use homologous recombination in many ways. They’ll probably be able to use it to correct genes, and that was what I hoped to be able to do 30 years ago. It’s delightful.

**Gitschier:** The other thing I noticed in your Nobel Prize speech was your repeated emphasis: “Pay attention, students!”

**Smithies:** Yes. Every five years we have to write up a review of whether we can go on working at the University. And I put down that though I would like to go on doing experiments, what motivates me most nowadays is to try to convey to young scientists the things that will help them in their careers.

I try to help students think about their work, knowing when to give up and when to change and when to say, “I don’t like what I’m doing, I’m not going to go on doing this the rest of my life.” To help people to make those decisions.

I’ve found that whenever I give this sort of lecture, one or two students in the audience react like this: [he changes his facial expression to eyes-wide, alert], meaning that they are probably students who are not liking what they are doing. I tell them that if you aren’t doing what you like, go to your advisor and ask for a different problem, and if your advisor can’t give you a different problem, change your advisor. In an audience of two or three hundred students, there will always be one or two graduate students who are not happy with their work, and they don’t know what to do.

**Gitschier:** They feel trapped.

**Smithies:** Yeah, they feel trapped. And I say, if you don’t like science after a while, go on and play the guitar, or go on and be a gardener. Do something that you enjoy. Don’t tie yourself to something you don’t like.

**Gitschier:** That’s great advice.

**Smithies:** Yes, “Don’t tie yourself to something you don’t like,” is a good ending.

## Note Added in Proof

The University of North Carolina administration became aware of comments in this interview regarding its decision to terminate archiving Smithies' notebooks. They have reached out to Smithies and plan to take up the project again.

